# Negotiating Reproductive Identity After Cancer: A Qualitative Systematic Review of Female Survivors' Post‐Treatment Fertility Experiences

**DOI:** 10.1111/hex.70811

**Published:** 2026-10-21

**Authors:** Yijun Wang, Margo Louise Turnbull, Jeffry Oktavianus, Xiaoyan I. Wu

**Affiliations:** ^1^ Department of English and Communication The Hong Kong Polytechnic University Kowloon Hong Kong SAR China

**Keywords:** cancer survivors, fertility decision‐making, qualitative research, reproductive concerns, reproductive health, survivorship, systematic review

## Abstract

**Background:**

Fertility concerns are common among female cancer survivors and are shaped by sociocultural influences that extend beyond biomedical outcomes. Previous systematic reviews have consolidated evidence on fertility‐related distress but have tended to operate within clinical or descriptive frameworks. Although identity‐related findings have emerged in existing reviews, they received limited conceptual attention.

**Objective:**

Guided by the concept of reproductive identity, this review systematically synthesises qualitative research on how female cancer survivors experience, negotiate, and reconstruct their reproductive identities within the broader context of post‐cancer survivorship.

**Methods:**

A systematic search was conducted in eight databases for qualitative studies published in English and Chinese between January 2010 and February 2026. The review followed PRISMA guidelines and was prospectively registered in PROSPERO (CRD42024604353). Data were synthesised using thematic synthesis, with coding conducted inductively before the reproductive identity framework was applied as an interpretive lens.

**Results:**

Thirty studies met the inclusion criteria. Three analytical themes were identified: (1) macro‐level discourses and structures shape reproductive identity—fertility is positioned as secondary to survival within cancer care and constrained by sociocultural motherhood narratives; (2) micro‐level relational dynamics and internal processes shape reproductive identity—fertility concerns are navigated within intimate relationships and through ongoing (re)working of reproductive selfhood; and (3) lived experience informs the reimagining of macro‐level care—participants' experiences of structural constraints generated insight into how care systems could better support reproductive identity across survivorship.

**Conclusion:**

The findings of this review highlight that post‐cancer fertility is a dynamic process of identity negotiation shaped by the interplay between macro‐level discourses and micro‐level relational and internal processes. This reinforces the importance of embedding reproductive identity literacy into survivorship care through proactive, clinician‐initiated fertility conversations and coordinated referral pathways that extend beyond the treatment phase.

**Patient and Public Contribution:**

Patient and public involvement were not formally incorporated into this review. However, the first author is a female cancer survivor with lived experience of fertility preservation, whose patient perspective contributed to the development of the research, the interpretation of findings and the framing of implications for survivorship care.

## 1 Introduction

Incidence rates of cancer among individuals of reproductive age (15–49 years, as defined by the World Health Organisation [1]) have risen significantly in recent decades [2, 3]. These increases have occurred alongside innovations in fertility preservation technologies and advances in treatments that have improved survival rates [4]. This is particularly evident in haematological malignancies and early‐stage breast cancer, where long‐term survivorship is increasingly common but treatments remain highly gonadotoxic [5, 6]. Recent research in oncofertility has highlighted the complexity of post‐treatment fertility and identified links between fertility‐related distress and lowered quality of life [7, 8, 9]. Although these connections are evident across genders, women report significantly higher levels of distress than men [10, 11]. Additionally, accessing fertility preservation services does not necessarily translate into utilisation of cryopreserved material. Recent systematic reviews indicate that only 5%–10% of women returned to use their preserved reproductive material [12, 13], with many viewing cryopreservation as an '*insurance policy*' (p. 31) for their reproductive future rather than a definitive plan [14].

A growing body of quantitative and qualitative research has drawn attention to the psychological, relational, and sociocultural dimensions of fertility after cancer [15, 16, 17, 18, 19, 20], establishing fertility‐related distress as a recognised concern in survivorship care. Previous systematic reviews have built on this foundation, progressively refining evidence on post‐cancer fertility, though primarily within frameworks oriented toward clinical outcomes and psychosocial experiences. Logan et al. [21] established the persistence of fertility‐related psychological distress across the cancer trajectory, providing a foundation for subsequent work. Dong et al. [10] broadened this evidence base by mapping reproductive concerns across gender, age, and treatment stages, while Yoshida et al. [22] narrowed the focus to post‐treatment survivorship and identified a wider range of psychosocial needs. Most recently, Zhang et al. [23] examined women's fertility decision‐making processes and reported identity‐related findings, but these were supported by limited evidence and fell outside the review's analytical focus. These reviews have been productive in documenting what survivors experience but are not structured to examine how those experiences intersect with broader processes of identity negotiation and reconstruction. Notably, identity‐related findings have nonetheless emerged in recent syntheses [22, 23], suggesting that these dimensions are present in the primary literature but have yet to be examined through a dedicated conceptual framework.

To address this gap, this review takes up Athan's [24] concept of reproductive identity (RI) as a guiding framework. Athan [24] conceptualised it as a superordinate developmental construct that evolves across the life course and exists on a non‐binary spectrum. She further proposed that RI is shaped and reshaped through the interplay between macro‐ and micro‐level forces [24]. At the macro level, sociocultural norms, institutional structures, and dominant narratives prescribe what a ‘normal’ reproductive life course should look like [25]; at the micro level, individuals personally (re)interpret their reproductive circumstances and navigate these within intimate and relational contexts [24]. In this formulation, RI emerges through the dialogue between these levels, as individuals encounter, internalise, or resist macro‐level scripts in the process of making meaning of their own reproductive experiences. The concept [24] has recently been expanded to the study of reproductive identity among women undergoing IVF [26], and to frame the development of the Reproductive Identity Questionnaire [27]. By applying the RI framework to a qualitative synthesis of female cancer survivors' post‐treatment fertility experiences, this review offers a perspective absent from previous systematic reviews: an integrative, developmental lens examining how macro‐ and micro‐level influences shape survivors' RI. To do this, we address the following research questions: (a) How are macro‐level narratives reproduced in female cancer survivors' experiences of post‐treatment fertility? (b) What micro‐level relational and personal processes are evident in experiences of RI shaping? (c) What insights about macro‐level care structures emerge from survivors’ experiences of negotiating RI?

## 2 Methods

### 2.1 Search Strategy

This systematic review was registered prospectively with PROSPERO (CRD42024604353) and is reported in accordance with the Preferred Reporting Items for Systematic Reviews and Meta‐Analysis (PRISMA 2020) guidelines [28]. Searches were initially run between 30 October and 9 November 2024 and updated on 12 February 2026. Searches included eight databases: Web of Science, PubMed, Medline, Embase, CINAHL Complete, PsycINFO, CNKI and SinoMed. Studies published in English and Chinese were included to capture diverse perspectives from international literature as well as culturally specific experiences documented in Chinese contexts. Search terms in both English and Chinese were developed in consultation with a specialist librarian, combining terms across five conceptual blocks (cancer, fertility, female population, psychological and social factors, and survivorship period) using Boolean operators. Chinese search terms were adapted to the indexing conventions of Chinese‐language databases. Search terms were applied consistently across all databases; database‐specific filters such as publication type and date range were applied where available. To ensure relevance to contemporary survivorship and advancements in fertility preservation and care, date limits were set on the searches, and studies published from 2010 onwards were included. The full search strategy is detailed in Supporting Information: Appendix A. Additional records were identified through manual backward and forward citation searching.

### 2.2 Inclusion and Exclusion Criteria

This review included qualitative studies to capture the depth and complexity of personal fertility experiences that may not be foregrounded in quantitative research. Studies were included if research participants were female and were within the post‐treatment survivorship period, defined as the phase following completion of primary cancer treatment (surgery, chemotherapy and/or radiotherapy), encompassing early follow‐up through long‐term survivorship. Age eligibility was operationalized at the study level: studies were eligible when participants were aged 18–50 years at diagnosis, treatment, or data collection, or when the sample was predominantly within this range and findings for eligible participants could not be meaningfully separated from the overall qualitative dataset. Where a small number of participants were older than 50 at interview, studies were retained only if the fertility‐related experience under investigation occurred during reproductive age and if the sample was predominantly within the eligible range. Findings explicitly attributable only to participants older than 50 were not extracted for synthesis. The age range was informed by the WHO definition of reproductive age (15‐49 years) [1], within which fertility‐related concerns are most pronounced [29]: the lower limit was adjusted to 18 to reflect standard adult research ethics requirements, while the upper limit was extended to 50 to account for variation in menopausal onset [30]. Additionally, research questions of the studies included needed to relate to the exploration of psychological or social dimensions of post‐cancer fertility, such as emotional well‐being, partner and family dynamics, societal expectations, and peer support. Studies with mixed‐gender or mixed‐role samples (e.g., dyadic studies including partners, or studies involving healthcare providers) were eligible if non‐female participant data could be clearly identified and excluded from the synthesis.

Studies were excluded if they focused solely on male survivors, samples whose average age exceeded 50 years, experiences limited to the diagnostic or acute treatment phase (i.e., pre‐survivorship periods), or utilised quantitative and/or mixed methods. Research addressing purely biomedical outcomes (e.g., in vitro fertilisation success rates or reporting of hormonal levels) was also excluded.

### 2.3 Study Selection

Search results were downloaded to EndNote, and duplicates were removed. The first author (YW) conducted title and abstract screening of all studies in consultation with the second author (MT). The fourth author (XW) reviewed those published in Chinese. All full‐text articles were screened by two authors (YW and MT screened those in English, YW and XW screened Chinese publications). Any disagreements about inclusions were resolved through discussion.

### 2.4 Data Extraction

Data were extracted from all included articles by YW. Extracted data included the first author, title, publication year, country, patient characteristics, data collection and analysis methods, phenomena of interest, and the primary findings (see Supporting Information: Appendix B). MT independently reviewed the extracted data to verify accuracy and completeness, and any discrepancies were resolved through discussion.

### 2.5 Data Synthesis

Thematic synthesis [31] was used to analyse and integrate the findings of the included studies, following three stages. In the first two stages, coding and the development of descriptive categories proceeded inductively, without reference to the RI framework or any other predetermined theoretical structure. Initially, a preliminary coding framework was developed through collaborative work between YW [16, 32, 33, 34, 35, 36, 37, 38, 39, 40, 41, 42, 43, 44] and MT [17, 45, 46, 47, 48, 49, 50] who independently coded their assigned studies (without overlap), refining the framework through ongoing discussion. This iterative process resulted in six inductively derived coding categories: fertility and medical interactions; fertility and emotions; fertility and identity of self; fertility and relationships (partner, family, peers); fertility and values/beliefs; and the event of pregnancy. These categories formed the basis of a structured codebook (Supporting Information: Appendix C), which was then used to guide subsequent coding by JO (English‐language studies [51, 52, 53, 54]) and XW (Chinese‐language studies [55, 56, 57, 58, 59]). All authors independently coded their assigned articles, and progress was regularly discussed by the authorship group. To assess intercoder consistency and enhance the trustworthiness of the synthesis in accordance with qualitative research approaches [60], 4 of the 30 included studies (~13.3%) were double‐coded.

During the iterative coding process, it became evident that the six categories were not discrete but conceptually intertwined. To move beyond description and examine these interconnections analytically, Athan's [24] RI framework was introduced in the third stage as an interpretive lens. The framework's macro–micro analytical architecture provided a means of reorganising the descriptive categories, as individual categories often contained both macro‐ and micro‐level elements; delineating these dimensions could provide insights not otherwise evident [61]. Through this reorganisation, analytical themes and subthemes were identified and mapped to the framework's macro‐ and micro‐level dimensions (refer to the summary in Table 1).

**Table 1:** Mapping of included studies to reproductive identity framework [24].

No.	Study	Country	Macro‐level reproductive identity (RI) dimensions	Micro‐level RI dimensions	Notes on RI applicability
1	[47]	UK	Information/communication gaps	Disclosure/dating concerns; Contradictory/conditional emotions; Emotional response	Primarily micro‐level; Limited macro engagement
2	[45]	Australia	Survival discourse; Motherhood narratives; Information/communication gaps	Disclosure/dating concerns; Redefining selfhood; Emotional response	Strong fit across both levels
3	[48]	US and Canada	Information/communication gaps; Structural barriers	Emotional response; Redefining selfhood	Strong fit across both levels
4	[49]	Mexico	Survival discourse; Structural barriers; Information/communication gaps	Minimisation/suppression; Emotional response; Relational dynamics; Disclosure/dating concerns	Strong fit across both levels
5	[51]	US	Structural barriers; Information/communication gaps	Emotional response; Contradictory/conditional emotions	Strong fit across both levels
6	[53]	US	Information/communication gaps; Structural barriers	Emotional response	Primarily macro‐level; Limited micro engagement
7	[50]	UK	Survival discourse; Structural barriers	Relational dynamics; Emotional response	Strong fit across both levels
8	[16]	Canada	Motherhood narratives	Emotional response; Contradictory/conditional emotions; Redefining selfhood	Primarily micro‐level; Limited macro engagement
9	[34]	US	Information/communication gaps; Motherhood narratives	Disclosure/dating concerns; Redefining selfhood; Emotional response	Strong fit across both levels
10	[17]	Australia	Survival discourse; Motherhood narratives	Relational dynamics; Emotional response; Redefining selfhood	Strong fit across both levels
11	[46]	US	Survival discourse; Information/communication gaps	Emotional response; Redefining selfhood	Strong fit across both levels
12	[35]	UK	Survival discourse; Structural barriers; Information/communication gaps	Emotional response	Primarily macro‐level; Limited micro engagement
13	[36]	US	Information/communication gaps; Structural barriers	Relational dynamics; Emotional response; Contradictory/conditional emotions	Strong fit across both levels
14	[32]	Australia	Motherhood narratives; Information/communication gaps	Emotional response	Primarily macro‐level; Limited micro engagement
15	[54]	UK	Information/communication gaps; Structural barriers	Emotional response	Primarily macro‐level; Limited micro engagement
16	[52]	UK	Motherhood narratives; Information/communication gaps	Disclosure/dating concerns; Emotional response; Relational dynamics	Strong fit across both levels
17	[37]	Japan	Motherhood narratives; Familial expectations	Relational dynamics; Redefining selfhood	Strong fit across both levels
18	[38]	Australia	Motherhood narratives	Relational dynamics; Emotional response	Primarily micro‐level; Limited macro engagement
19	[39]	US	Motherhood narratives; Structural barriers	Relational dynamics; Emotional response; Contradictory/conditional emotions	Strong fit across both levels
20	[57]	China	Familial expectations	Relational dynamics; Emotional response	Primarily micro‐level; Limited macro engagement
21	[55]	China	Information/communication gaps	Contradictory/conditional emotions; Relational dynamics; Emotional response	Primarily micro‐level; Limited macro engagement
22	[58]	China	Motherhood narratives; Information/communication gaps	Emotional response; Relational dynamics	Strong fit across both levels
23	[59]	China	Information/communication gaps; Familial expectations	Emotional response; Relational dynamics; Contradictory/conditional emotions	Strong fit across both levels
24	[40]	China	Information/communication gaps; Structural barriers	Emotional response; Redefining selfhood	Strong fit across both levels
25	[56]	China	Motherhood narratives; Familial expectations	Emotional response; Relational dynamics	Strong fit across both levels
26	[44]	China	Motherhood narratives; Familial expectations	Disclosure/dating concerns; Redefining selfhood; Emotional response	Strong fit across both levels
27	[41]	UK	Structural barriers; Information/communication gaps	Emotional response; Redefining selfhood; Relational dynamics	Strong fit across both levels
28	[42]	South Korea	Survival discourse; Structural barriers; Familial expectations; Information/communication gaps	Relational dynamics	Primarily macro‐level; Limited micro engagement
29	[43]	Canada	Structural barriers; Motherhood narratives	Emotional response; Redefining selfhood	Strong fit across both levels
30	[33]	China	Motherhood narratives; Familial expectations; Structural barriers	Relational dynamics; Disclosure/dating concerns; Emotional response; Redefining selfhood	Strong fit across both levels

*Note:* “Notes on RI applicability” summarises the overall fit of each study's findings with the framework's analytical architecture. Of the 30 included studies, 20 contained findings that engaged with both macro‐ and micro‐level dimensions.

### 2.6 Critical Appraisal

All included studies were appraised for methodological quality using the CASP Qualitative Checklist [62]. The purpose of this appraisal was to systematically evaluate the rigour of each study and to inform the interpretation of findings, rather than to exclude studies from the synthesis. For the English‐language studies, MT appraised seven studies (23.3%) and YW appraised the remainder; for the Chinese‐language studies, XW appraised six studies (20%) and YW appraised one. The appraisal outcomes and their implications for confidence in the synthesized themes are reported in Section 3.3.

### 2.7 Reflexivity Statement

The authorship team includes individuals of diverse nationalities, language backgrounds, genders, and lived experiences. The first author, a female cancer survivor with lived experience of fertility preservation, brought an insider perspective that informed the research focus and interpretation of findings. All other members of the research team are experienced researchers with extensive backgrounds in health‐related topic areas. The research team engaged in regular reflexive discussions throughout the coding and synthesis process to examine how their respective positions may have shaped the identification and interpretation of themes.

## 3 Results

### 3.1 Search Results

A total of 2954 records were identified through database searching, and 2172 duplicates were removed. After screening 782 titles and abstracts, 39 articles were retrieved for full‐text review and assessed for eligibility. Of these, 18 studies were included. An additional 54 records were identified through citation searching, of which 12 met the inclusion criteria. In total, 30 studies were included in the review (Figure 1). Of these, 23 were published in English and seven in Chinese. The Chinese‐language studies, all conducted in mainland China, explored fertility‐related concerns and decision‐making among survivors of breast, thyroid, kidney and gynaecological cancers.

**Figure 1:**
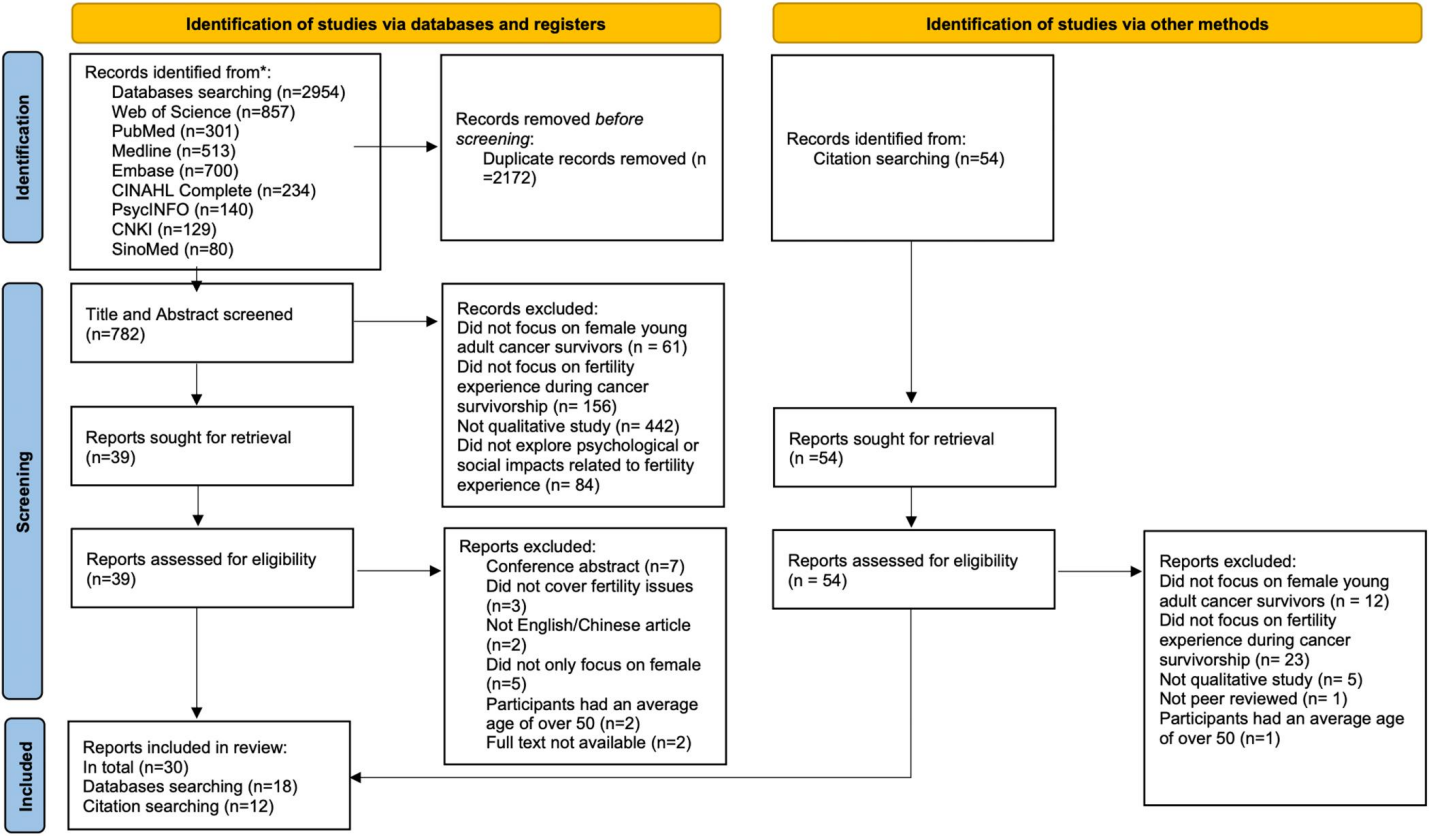
PRISMA diagram.

### 3.2 Included Study Characteristics

The included studies were conducted in diverse geographical locations, namely Australia, Canada, China, Japan, Mexico, South Korea, the UK and the United States. Two pairs of articles drew on shared samples from two separate projects [51, 52, 53, 54], but examined different aspects of fertility experiences. Participants from overlapping samples were counted once in the total. One study [34] included a male participant, who was excluded from analysis, and two [39, 57] used dyadic designs with heterosexual couples; only the female participants' data were included. One study [42] included both patient and healthcare provider perspectives; only patient data were analysed. After accounting for these overlaps and exclusions, the total number of unique female research participants was 477, with varied relationship and parenting statuses reported. Breast cancer was the most reported diagnosis, followed by cervical, ovarian, haematologic, and brain cancers. Other diagnoses included gestational trophoblastic neoplasia and various childhood cancers. Study characteristics are summarised in Table 2.

**Table 2:** Summary characteristics and key findings of the studies.

Number	Study (year)	Country	Aim	Methodology & analysis method	Sample size and age*	Cancer type	Key findings
1	Lloyd et al. [47]	UK	To explore women's experiences 1–10 years following a radical vaginal trachelectomy and describes the impact on health, sexuality, fertility and perceived supportive care needs.	Semi‐structured interviews, a descriptive phenomenological approach	*N* = 12 women; Age at surgery: 29–45 years	Cervical cancer	• Positive psychosocial growth alongside long‐term concerns (fear of recurrence, sexual discomfort, pregnancy anxiety) • Reliance on clinical staff, peers, and online forums for support • Information gaps and unmet needs for continuity of care and counselling
2	Dryden et al. [45]	Australia	To explore younger women's construction of their fertility post‐cancer, and their discussions of fertility with healthcare professionals.	Semi‐structured interviews, Foucauldian Discourse Analysis	*N* = 8 women; Age at interview: 18–26 years	Leukaemia, brain, breast, lymphoma, sarcoma	• Discursive positions: accepting, resisting, internalising motherhood mandate • Emotional impacts: fear, inadequacy, relational insecurity • Dissatisfaction with provider communication and decision‐making support
3	Ruddy et al. [48]	US and Canada	To explore existing supports that are important during diagnosis and treatment and the unmet needs for information and support in young women with breast cancer.	Semi‐structured interviews	*N* = 20 women; Age at diagnosis: ≤ 42 years	Breast cancer	• Faith, spirituality, and family as main supports • Limited peer connections and inadequate support for families • Unmet informational needs on fertility, menopause, relationships, work, and survivorship
4	Hubbeling et al. [49]	Mexico	To describe the psychosocial needs of young breast cancer survivors in Mexico at 5 or more years of survivorship, identifying areas of focus for early interventions.	Semi‐structured interviews; Thematic analysis	*N* = 25 women; Age at diagnosis: 31–40 years; Age at interview: 37–53 years	Breast cancer	• Minimisation of fertility concerns • Persistent body image disturbance • Barriers to employment during survivorship • Impact on family relationships and networks • Unmet psychological and informational needs
5	Benedict et al. [51]	US	To examine adolescent and young adult female (AYA‐F) survivors' experiences and decision‐making related to family building after cancer.	Semi‐structured interviewshypothesis coding and grounded theory coding methods	*N* = 25 women; Age at interview: 15–39 years (mean 29)	Breast, gynaecological, haematologic, colorectal, and other rare cancers	• Uncertainty (medical, personal, social, financial) • Emotional and cognitive reactions • Coping behaviours • Decision‐making processes in family building
6	Benedict et al. [53]	US	To examine AYA‐F survivors' unmet needs and recommendations for care to address fertility/family‐building in post‐treatment survivorship.	Semi‐structured interviewsgrounded theory methods	*N* = 25 women; Age at interview: 15–39 years (mean 29)	Breast, gynaecological, haematologic, colorectal, and other rare cancers	• Survivors emphasised need for improved patient–provider communication • Greater access to informational, emotional, peer, and financial support • Telehealth/online resources acceptable for initial contact • Preference for in‐person discussions on sensitive topics
7	Sobota, Ozakinci [50]	UK	To apply Common‐Sense Model and shared decision‐making model to explore experiences of treatment decision‐making women of reproductive age who were diagnosed with gynaecological or breast cancer and the influence of fertility issues and fears of cancer progression and recurrence.	interviewsthematic analysis	*N* = 24 women; Age at interview: 31–45 years	Gynaecological or breast cancer	• Awareness of infertility risks • Balancing cancer treatment versus fertility considerations • Evaluation and consequences of treatment decisions • Influences from cancer type, provider attitudes, relationship status, and recurrence fears
8	Vanstone et al. [16]	Canada	To better understand women's experiences of pregnancy and the postpartum period following cancer treatment through a qualitative analysis.	Semi‐structured interviewgrounded theory approach	*N* = 10 women; Age at interview: 35–40 years	Non‐Hodgkin's lymphoma; leukaemia; breast cancer	• Mixed emotions in perinatal period (happiness vs. anxiety) • Grief over fertility loss and conditional joy during pregnancy • Frustration at lack of post‐cancer pregnancy resources • Hope during transition to motherhood
9	Sira et al. [34]	US	To understand the experiences of childhood cancer survivors who are faced with infertility as a late treatment effect while reconstructing their identity	Semi‐structured interviews, guided by the theoretical framework of identity development	*N* = 5 women, *N* = 1 man; Age at interview: 18–29 years	Diverse cancers (completed treatment during youth)	• Uncertainty about future identity • Challenges in intimacy and communication • Reconstruction of identity through new roles • Coping via family support
10	Rowlands et al. [17]	Australia	To explore the impact of gynaecological cancer on women's expectations for motherhood, their concerns regarding their fertility, and how they negotiated these concerns in the survivorship context.	Semi‐structured interviews, Thematic discourse analysis, from a material‐discursive‐intrapsychic approach	*N* = 42 womenAge at diagnosis: 21–44 years (mean 34); Age at interview: 27–53 years (mean 39)	Gynaecological cancer	• Fertility concerns negotiated within conflicting discourses: pronatalism versus survivorship focus • Fertility‐related decision‐making socially and emotionally complex
11	Gorman et al. [46]	US	To explore how young women make cancer treatment decisions and the role of fertility concerns in that process.	Open‐ended telephone interviews and used cross‐case, inductive analysis	*N* = 20 women; Age at interview: 26–38 years	Breast cancer	• Survival prioritised over fertility, especially among younger/childless women • Conflicting professional advice heightened fear • Regret over missed preservation opportunities and lack of transparent discussions
12	Lee et al. [35]	UK	To investigate women's responses to being told that treatments affected their fertility and how their interactions with health services impacted on their experiences.	3 focus groups using a flexible interview structureThematic analysis	*N* = 24 women; Age at diagnosis: 23–39 years	Breast cancer	• Women felt deprived of reproductive choices by cancer, treatment, and health system • Desire for earlier referral to fertility specialists • Retrospective wish for more informed decisions
13	Gorman et al. [36]	US	To explore the fertility and parenthood concerns of reproductive‐age female cancer survivors and how they make parenthood decisions.	7 focus groups cross‐case, inductive analysis	*N* = 22 women; Age at interview: 18–34 years	Broad range of cancers, most commonly lymphoma, breast, and thyroid.	• Hope mixed with anxiety and frustration over lost control • Strong desire for fertility information and continuity of care • Relationship challenges and complex parenthood decisions
14	Kirkman et al. [32]	Australia	To learn about women's experiences of fertility‐related cancer care	In‐depth qualitative interviews, thematic analysis	*N* = 10 women; Age at diagnosis: 25–41 years; Age at interview: 26–45 years	Breast cancer	• Fertility highly valued regardless of parental status • Experiences of fertility care varied (supportive to dismissive) • Emphasised clear, individualised, assumption‐free communication
15	Corney, Swinglehurst [54]	UK	To investigate in detail the fertility‐related experiences of young childless women with breast cancer, including the information they received, the fertility preservation options given, and the dilemmas they faced.	interviews, thematic analysis	*N* = 19 women; Age at diagnosis: 20–41 years; Age at interview: 24–44 years	Breast cancer	• Wide variation in fertility information provision • Only half offered fertility preservation pre‐treatment • Common worries about fertility and child health post‐treatment • Lack of emotional and informational support
16	Corney et al. [52]	UK	To explore the stressors and vulnerabilities of young single childless women with a first episode of breast cancer.	In‐depth semi‐structured	*N* = 10 women; Age at diagnosis: 27–41 years; Age at interview: 30–44 years	Breast cancer	• Key themes: relationship concerns, fertility dilemmas, perceptions of support • Single women felt vulnerable, feared romantic rejection • Reported inadequate emotional and practical support
17	Komatsu et al. [37]	Japan	To explore the experience of fertility preservation with radical trachelectomy from the perspective of women with cervical cancer.	Interviews, Grounded Theory methodology with a theoretical framework of symbolic interactionism.	*N* = 15 women; Age at surgery: 25–38 years	Cervical cancer	• Trachelectomy restored sense of femininity threatened by cancer • Identity reconstruction occurred through self–other interactions • Individual meanings and perspectives varied
18	Standen et al. [38]	Australia	To explore attitudes to conception in partners and young women, following fertility preserving treatment for gynaecologic cancers.	Semi‐structured interviews, thematic analysis	*N* = 14 women, *N* = 2 partners; Mean age at interview: 33 years	Early‐stage gynaecologic/borderline ovarian tumours (fertility‐sparing surgery)	• Emotions at diagnosis and information received • Fertility discussions and factors affecting childbearing • Partner roles in decision‐making and relationship pressures • Postoperative regrets regarding treatment choices
19	Gorman et al. [39]	US	To explore how YABCS and their partners appraised and managed family building after cancer to inform approaches to support these couples.	Semi‐structured interviews, thematic analysis	*N* = 25 women, *N* = 25 male partners; Age at interview: 18–39 years	Breast Cancer	• Open communication between partners • Mismatched priorities and limited reproductive choices • Emotional and instrumental support • Concerns about social expectations, timing, and financial barriers
20	Zhou et al. [57]	China	To explore the fertility‐related psychological distress of young female renal cancer patients from a dyadic perspective, examine the role of their spouses.	Semi‐structured interviews; Colaizzi's 7‐step analysis method	*N* = 13 women, *N* = 13 male partners; Age at interview: 24–37 years	Kidney cancer	• Fertility uncertainty caused negative emotions in patients • Spouses' reactions shaped patient experiences • Couples faced challenges together • Spousal support facilitated active coping
21	Qiu et al. [55]	China	To investigate the emotional experiences and support needs of young breast cancer patients who have gone through childbirth after being diagnosed with breast cancer.	Semi‐structured interviews, phenomenological study; Colaizzi's 7‐step analysis method	*N* = 12 women; Age at interview: 24–40 years	Breast cancer	• Mixed emotions during conception and childbirth (anxiety, joy, fear) • Support needs: professional guidance, family involvement, peer connection
22	Li, Qiang [58]	China	To explore the real experiences of young female breast cancer patients who have undergone radical mastectomy.	Semi‐structured interviews, phenomenological study; Colaizzi's 7‐step analysis method	*N* = 12 women; Age at interview: 26–35 years	Breast cancer	• Body image disturbance, sexual and fertility concerns after mastectomy • Role changes in family and career uncertainty • Persistent unmet psychological and informational needs
23	Tang et al. [59]	China	To understand the real experiences of fertility concerns among young female thyroid cancer patients after diagnosis and surgery.	Semi‐structured interviews, phenomenological study; Colaizzi's 7‐step analysis method	*N* = 13 women; Age at interview: 23–28 years	Thyroid cancer	• Worries and self‐blame • Desire for communication and support • Need for scientific fertility knowledge
24	An et al. [40]	China	To explore the real decision‐making experience of fertility preservation in young patients with early breast cancer.	Semi‐structured interviews, phenomenological study; Colaizzi's 7‐step analysis method	*N* = 15 women; Age at interview: 29–38 years	Breast cancer	• Concerns • Individualised and dynamic reproductive decisions • Decision conflict • Demand for professional help in decision making.
25	Wang et al. [56]	China	To explore the current state of fertility concerns and the real psychological experiences of female breast cancer patients of reproductive age.	Semi‐structured interviews, phenomenological study; Colaizzi's 7‐step analysis method	*N* = 12 women; Age at interview: 24–40 years	Breast cancer	• Self‐concerns regarding fertility • Concerns about children's health and caregiving • Strong demand for fertility‐related information • Negative emotional experiences due to childlessness
26	Yao et al. [44]	China	From a life course perspective, deeply investigate the role of marriage and fertility decision‐making in the life course reconstruction of gynaecological cancer survivors.	Semi‐structured interviews	*N* = 9 women; Age at interview: 25–40 years	Gynaecological cancer	• Dual pressure: Health challenges and societal role expectations • Coping process: The interplay between life status and marriage/fertility choices • Fertility autonomy: A key stage in reconstructing the life course
27	Singh et al. [41]	UK	To explore women's experience of the period after completion of cancer treatment for gestational trophoblastic neoplasia (GTN).	Semi‐structured interviews; reflective thematic analysis	*N* = 22 women; Age at interview: 23–54 years (mean 33)	Gestational trophoblastic neoplasia (GTN)	• Treatment completion as a ‘double‐edged sword’: relief coupled with loss of structured support • Grief over pregnancy loss and anxiety about future pregnancy and recurrence • Coping through re‐evaluation of priorities and peer connections • Gaps in post‐treatment follow‐up, peer support, and counselling
28	Han et al. [42]	South Korea	To identify the barriers to and facilitators of shared decision‐making implementation in fertility preservation for female patients with cancer.	Semi‐structured interviews; directed content analysis guided by implementation science framework	*N* = 16 female patients (age 28–41 years); *N* = 7 healthcare providers	Predominantly breast cancer	• Barriers to FP shared decision‐making: limited accessibility, time constraints, insufficient interdisciplinary communication • Patient‐level barriers: limited knowledge, psychological burden, passive attitudes • Sociocultural and organisational influences: family decision‐making dynamics, high cost, policy limitations • Facilitators: family support, multidisciplinary care, social shift towards proactive FP
29	Banser et al. [43]	Canada	To describe the challenges female adult childhood cancer survivors experienced while navigating assisted reproductive technology and family‐building options.	Semi‐structured interviews; Interpretive Description; interpretive thematic approach and constant comparative techniques	*N* = 15 women; Age at interview: 25–48 years (mean 35.6); Mean age at diagnosis: 9.5 years	Various childhood cancers (Wilms tumour, leukaemia, lymphoma, Ewing's sarcoma, brain/CNS tumour, rhabdomyosarcoma)	• Unexpected impaired fertility and grief over loss of reproductive identity • Unsupportive and fragmented healthcare encounters • Adoption inaccessible for cancer survivors; some sought ART internationally • Significant financial strain from ART costs and limited public funding
30	Xu et al. [33]	China	To explore the reproductive concerns experienced by young breast cancer survivors' post‐treatment and the factors influencing their perceptions.	Semi‐structured interviews; descriptive phenomenological design; Colaizzi's method	*N* = 18 women; Age at interview: 27–39 years	Breast cancer	• Multiple emotional burdens interwoven with fertility concerns and societal pressure • Concerns about reproduction risks and childrearing dilemmas • Reproduction valued for identity, rehabilitation motivation, and family integrity • Support needs from family, peers, and multidisciplinary professionals

*Age ranges reflect the information reported in each original study. Where both age at diagnosis and age at interview were available, both are reported.

### 3.3 Quality of Included Studies

Using the CASP assessment tool, 23 of the 30 included studies were assessed as high quality (score > 9) and seven as moderate quality (score 7–9). None was considered low quality. All studies provided clear statements of aims and findings and used appropriate qualitative methodologies and research designs (Supporting Information: Appendix D). The most common limitation related to reflexivity (Criterion 6): only five studies [17, 33, 43, 49, 50] provided an explicit account of the relationship between researchers and participants. Seven studies received an unclear rating on ethical considerations (Criterion 7): four Chinese‐language studies [40, 44, 56, 57] did not include explicit statements of ethical approval, though all provided detailed accounts of informed consent procedures, and three English‐language studies [35, 36, 47] provided limited discussion of ethical issues. Recognising the limitations of applying quality appraisal tools to produce consistent judgements, no studies were excluded based on the CASP assessment. The identified limitations related to reporting conventions rather than methodological conduct, and each analytical theme was supported by multiple studies, including those rated as high quality across all CASP criteria.

### 3.4 Findings

Three analytical themes emerged from the data analysis: (1) macro‐level discourses and structures shape reproductive identity; (2) micro‐level relational dynamics and internal processes shape reproductive identity; and (3) lived experience informs the reimagining of macro‐level care. The distribution of included studies across themes and subthemes is presented in Table 3. Each subtheme was supported by between 19 and 22 of the 30 included studies, with each study contributing to at least two subthemes. Quotations from Chinese‐language studies were translated into English by YW and checked by XW.

**Table 3:** Distribution of included studies across themes and subthemes.

No.	Study	Country	Theme 1: Macro‐level discourses and structures		Theme 2: Micro‐level relational dynamics and internal processes		Theme 3: Reimagining macro‐level care	Total
			S1: Fertility in cancer care	S2: Motherhood narratives	S1: Intimate relationships	S2: (Re)working of selfhood		
1	[47]	UK			✓	✓	✓	**3**
2	[45]	Australia	✓	✓	✓	✓		**4**
3	[48]	US and Canada	✓				✓	**2**
4	[49]	Mexico	✓	✓	✓	✓	✓	**5**
5	[51]	US	✓	✓		✓		**3**
6	[53]	US	✓				✓	**2**
7	[50]	UK	✓		✓			**2**
8	[16]	Canada		✓		✓	✓	**3**
9	[34]	US	✓	✓	✓	✓		**4**
10	[17]	Australia	✓	✓	✓		✓	**4**
11	[46]	US	✓	✓		✓		**3**
12	[35]	UK	✓			✓	✓	**3**
13	[36]	US	✓	✓	✓	✓	✓	**5**
14	[32]	Australia	✓	✓			✓	**3**
15	[54]	UK	✓			✓	✓	**3**
16	[52]	UK	✓	✓	✓		✓	**4**
17	[37]	Japan	✓	✓	✓	✓		**4**
18	[38]	Australia		✓	✓		✓	**3**
19	[39]	US	✓	✓	✓	✓		**4**
20	[57]	China		✓	✓	✓		**3**
21	[55]	China	✓		✓	✓	✓	**4**
22	[58]	China		✓	✓	✓	✓	**4**
23	[59]	China		✓	✓	✓	✓	**4**
24	[40]	China	✓			✓	✓	**3**
25	[56]	China		✓	✓	✓		**3**
26	[44]	China	✓	✓	✓	✓		**4**
27	[41]	UK		✓	✓	✓	✓	**4**
28	[42]	South Korea	✓	✓			✓	**3**
29	[43]	Canada	✓	✓		✓	✓	**4**
30	[33]	China	✓	✓	✓	✓	✓	**5**
	Total		22	22	19	22	20	105

*Note*. ✓ indicates that the study contributed findings to the theme/subtheme. n = number of studies contributing to each theme/subtheme. Total = number of themes/subthemes to which each study contributed. Higher frequencies do not imply greater thematic importance or qualitative significance.

#### 3.4.1 Macro‐Level Discourses and Structures Shape Reproductive Identity

Across the included studies, participants described how biomedical and sociocultural narratives shaped their fertility experiences at key points of decision‐making.

**Subtheme 1: The positioning of fertility within cancer care**

Research participants consistently described fertility as being positioned within their care as a low‐order concern across phases of diagnosis to survivorship. Research participants detailed that this positioning of fertility was evident in various care practices, including discussion by clinicians that implicitly or explicitly positioned fertility as dispensable in the context of cancer treatment [32, 36, 39, 42, 43, 50, 52]. In two studies [36, 45], for example, participants described that rather than presenting fertility and survival as concurrent considerations, clinicians often conveyed that fertility was a separate matter to be addressed only once treatment was complete. In some cases, this biomedical framing extended to redefine motherhood itself: clinicians drew on a logic of survival to suggest that being alive for one's existing or future children mattered more than the ability to bear them [17, 46, 50]. As one survivor recalled:

“He's [the oncologist] always said, which I agree…it's alright having a baby but you've got to be there, to be around to bring it up…It's quite a stark way of saying it.” [50]

During post‐treatment recovery, some survivors continued to encounter resistance or dismissal when they raised fertility‐related concerns. One woman said:

“After I was well, when I kept bringing up fertility and that I didn't want to do Tamoxifen [due to pregnancy being contraindicated during treatment], she kind of got frustrated with me… I think she felt like I was risking a lot.” [46].

According to research participants, this separation of ‘survival’ and fertility was reinforced in the design of institutional support and information systems, which acted as structural barriers. That is, fertility‐related information was rarely provided as part of oncology care. When it was available, participants described the information as vague, delayed, or inconsistent [17, 33, 34, 36, 40, 42, 43, 45, 46, 53]. In some studies [35, 36, 39, 40, 43, 45, 46, 48], participants described only learning about potential fertility preservation strategies after it was too late to act, which was viewed as a missed opportunity that carried long‐term emotional consequences [35, 36, 39, 40, 45, 46, 48]. As one participant reflected:

“I always kind of assumed that if I had difficulty conceiving or having kids, IVF would be an option, basically that I could throw money at the problem and it would help, but that wasn't the case.” [43]

Even among participants who were informed about fertility preservation options, structural barriers constrained their ability to act. Many described the interval between being told about preservation and the start of treatment as too short to meaningfully explore or pursue these options [35, 36, 37, 42, 46, 54]. Those who did proceed encountered a lack of standardised referral pathways and poor coordination across departments, barriers that persisted not only during the initial preservation process but also when participants later sought to use stored material or access assisted reproductive technologies (ARTs) [33, 36, 39, 42, 43, 52, 54]. The financial costs associated with these services posed an additional barrier to some participants [33, 36, 39, 42, 43, 50, 51]. Structural barriers to accessing fertility services were also influenced by assumptions about who deserved such support, with single women being either implicitly or explicitly discouraged or excluded from fertility preservation options [33, 40, 42, 44, 54]. In South Korea, access to embryo cryopreservation was restricted to married women, prompting one participant to rush into marriage registration to access it [42]. In Canada, restrictions on paid surrogacy and the limited availability of donor reproductive services led six of fifteen participants to seek ARTs internationally [43].

Across these accounts, many participants reported that they had come to perceive fertility as unimportant to their clinicians, or as an “*add‐on*” (p.7) [50] rather than an integrated component of cancer care [42, 43, 45, 54]. Over time, some women accepted this framing, feeling hesitant or ashamed to raise fertility‐related questions [17, 33, 42, 49, 55]. As one participant recounted:

“I felt embarrassed even asking my doctor, because I knew they would think, ‘You should be grateful to be alive… this is a place for treating illness, not for helping you have babies’” [55]

This tendency to view fertility as secondary to survival was described by participants across all geographical contexts represented in the included studies. This narrative was internalised by some participants who echoed these views own narratives, describing choices to forgo or delay reproductive plans as acts of responsibility, or expressing fears that pregnancy could trigger cancer recurrence [33, 35, 39, 40, 44, 46, 50, 51, 54]. As one woman reflected:

“I'd much rather be there and have one kid than not” [46]

It is important to note, however, that not all participants experienced the prioritisation of survival as externally imposed or as a source of ongoing tension. Some participants described choosing survival‐focused treatment as a genuine and unconflicted decision that did not generate lasting regret or unresolved fertility‐related distress, including those who had already completed their families, those who had been undecided about parenthood prior to cancer, and those who, over time, came to view survival itself as sufficient [37, 38, 39, 44, 46]. For these participants, the biomedical framing aligned with their own values.

**Subtheme 2: Sociocultural narratives about motherhood**

Participants also described their reproductive identities as shaped by sociocultural narratives positioning biological parenthood as central to femininity, social value, and adult womanhood. For many participants, the ability to bear children was perceived as a natural and integral aspect of being female [16, 17, 32, 33, 34, 37, 41, 43, 45, 46]. This was particularly pronounced among participants whose treatment involved the removal of reproductive organs or breasts, which represented a tangible disruption to embodied womanhood [17, 37, 49, 58]. In a study conducted in Japan, one survivor noted:

“The uterus is still a symbol of womanhood for many Japanese women.” [37]

Upon learning that their fertility had been compromised, participants described feeling “*shattered*”, “*heartbroken*” or “*like a failure as a woman in a way*” [17, 32, 33, 41, 43, 45, 46], with several noting that the emotional impact of potential infertility outweighed the trauma of the cancer diagnosis itself [32, 37]—for example, treatment will end but “*the fertility issue stays with you for life…it changes the whole course of your life*” [46].

Many participants also referred to a changing and intensifying awareness that their socially‐recognised reproductive window was narrowing, a tension particularly felt among those in their late twenties and thirties [36, 38, 39, 42, 51]. This pressure was compounded by comparisons with peers who were progressing through sociocultural milestones [39, 52]. For some, the inability to have children was interpreted not only as a personal loss but also as a social failure, particularly in relation to romantic partners and extended families [33, 37, 45, 56, 57]. In several accounts, fertility was framed as something to be *“given”* to a partner, and the inability to do so led to concerns about being abandoned or devalued [33, 39, 41, 43, 45, 57]. Unlike cancer, which was understood as an event beyond one's control, childlessness was perceived as something that invited social judgement and, in some accounts, explicit stigma [17]. As one participant reflected:

“Cancer is external…You can't help getting it…But when it comes to having a relationship, and having kids…that's something that you actively do. So if you don't have that, is there something wrong with you?” [45]

While these sociocultural narratives were present across all studies, studies conducted in the Chinese and Japanese contexts showed a distinct pattern in how familial expectations shaped fertility concerns [33, 37, 44, 56]. For example, data from China emphasised marriage as union and harmony between two families, and unmarried participants expressed particular concerns about how they would be perceived within extended family structures, fearing they would be “*looked down upon*” or that they would be “*holding back*” the other family [33, 44, 56, 59]. One participant said:

“My family wants me to have a baby…The atmosphere at home is tense, and I occasionally hear disparaging comments about my infertility from my elders and relatives. It's all because of my illness.” [33]

Similarly, in a study conducted in Japan, a participant expressed a strong desire to preserve her fertility to satisfy her husband's family, despite being advised to undergo a hysterectomy:

“My husband is the eldest child, and the only son in his family. So I wanted to have a child in consideration of my parents‐in‐law.” [37]

#### 3.4.2 Micro‐Level Relational Dynamics and Internal Processes Shape Reproductive Identity

Intimate relationships and women's own internal processes emerged as two key contexts in which fertility experiences were shaped.

**Subtheme 1: Fertility and the dynamics of intimate relationships**

For some single and childless participants, navigating romantic relationships was closely intertwined with fertility concerns [33, 34, 36, 37, 41, 44, 45, 52]. Participants' experiences were marked by fears of rejection and concerns about disclosure, with individuals reporting that they questioned not only how others would respond but also their own deservingness of intimacy [33, 36, 44, 47, 52]. As one participant reflected,

“That's so weird. It's like when do you tell someone, ‘Oh, by the way, like, I had cancer and there's a chance I won't be able to have a kid. Is that a problem?’” [36]

In a Chinese study, one participant said:

“Should I tell my boyfriend about this? Putting myself in his shoes, I don't think I could accept it if my girlfriend had one of her ovaries removed.” [44]

Some participants described withdrawing from dating altogether because of these compounding anxieties [34, 44, 49, 52]. A Chinese participant described her perception in the following way:

“Whether it will recur…it feels like a sword hanging over my head…If I want to enter an intimate relationship without any psychological burden, I think I would have to wait five years.” [44]

The absence of a partner also meant facing fertility‐related uncertainty without the sense of shared responsibility that partnered participants described. While some single participants received support from family members [34, 50], others noted that this did not carry the same emotional meaning [52].

For partnered participants, the presence of a partner meant that fertility‐related decisions were rarely faced alone. Many emphasised the importance of facing these decisions as a couple [17, 37, 38, 39, 50, 55]. As one participant explained:

“I so much felt like I needed that to be a joint decision. If the shit hits the fan basically…I couldn't ever have him saying ‘You kind of wanted this, you went off and did it'.” [50]

Some couples developed collaborative strategies such as attending medical appointments together, researching information jointly, and holding structured conversations [39, 55]. While positive partner support was described across all studies, its forms differed. In the English‐language literature, support was more frequently expressed through shared decision‐making [39, 50], whereas in the Chinese‐language studies, it was more often described in terms of companionship and practical caregiving, such as consulting with physicians on the participant's behalf or seeking encouraging information from online patient communities [55, 57].

Partner support, however, was not always experienced as open or collaborative. Some partners responded to fertility discussions by firmly prioritising survival in ways that foreclosed further conversation [17, 38, 39]. One participant recalled her partner saying:

“You need to have this surgery—there's no ifs, buts, or whats. You need to have this full radical hysterectomy,” [17]

and described that as *“the end of the story”* [17]. Other participants who felt they were limiting their partners' parenthood plans described feelings of guilt, echoing the sociocultural narratives that framed fertility as something to be “*given*” to a partner [41, 56, 57, 59]. In one study, this sense of guilt was compounded among participants who had lost a pregnancy through gestational trophoblastic neoplasia, where grief was intertwined with a sense of having failed their partners [41]. Some participants also described partners who avoided fertility‐related conversations altogether, intensifying emotional distress [38, 57]. One Chinese woman recalled:

“Sometimes I wanted to talk to my husband about how to plan for pregnancy after recovery, but he always changed the subject. I don't know what he's thinking. It made me so angry.” [57]

The strain of fertility uncertainty, combined with post‐treatment bodily changes, also affected physical intimacy. Participants described reduced sexual activity, physical avoidance, and emotional distance [33, 37, 41, 56, 57, 58]. For some, these pressures contributed to relationship breakdown [33, 38, 49].

**Subtheme 2: The (re)working of reproductive selfhood**

Many participants described intense emotional responses to the disruption of their reproductive possibilities as marking the beginning of an ongoing (re)working of reproductive selfhood. Across the included studies, participants described navigating fertility after cancer as emotionally draining and psychologically overwhelming [33, 35, 36, 37, 41, 43, 44, 51, 54, 59]. For some, the source of distress was not the loss of fertility itself but the loss of the possibility of choosing. As one Chinese participant expressed:

“What upset me was not that I desperately wanted a child, but that I had lost a choice.” [44]

This sense of uncertainty persisted beyond phases of treatment. Many participants placed their reproductive plans “*on hold*” due to the unknown impact of cancer treatments and struggled to make concrete plans without clear information [33, 54, 56]. The nature of this uncertainty also varied with cancer type and treatment pathway. Core concerns about foetal health, bodily safety during pregnancy, and disruption to reproductive plans were described across all cancer types, including those less commonly associated with direct reproductive harm, such as thyroid and kidney cancers [57, 59]. However, participants whose treatments carried a higher risk of permanent infertility described an additional layer of distress related to the potential loss of reproductive capacity itself and its broader identity implications. Those treated for childhood cancers often did not become aware of these impacts until years later, when they began to consider family‐building [34, 43].

Even those who had taken steps to preserve fertility often continued to feel unsure about their likelihood of achieving parenthood [51, 54]. As one participant noted:

“There's no guarantee that an embryo is going to turn into a baby.” [51]

For some, this period of uncertainty involved navigating what participants described as a “*new normal*” [41], a recognition that they could not return to who they were before, but a gradual process of re‐evaluating priorities and rebuilding a sense of self. As one participant reflected:

“I'm still figuring out who I am. I can't live my life how I was before…you can't go through this big thing and just nothing change.” [41]

Participants' reflections on their fertility‐related decisions and the pressures they faced changed over time and were (re)shaped by altered life circumstances and evolving desires [33, 39, 40, 43, 46, 51, 56]. Some reevaluated prior choices, regretting missed opportunities for fertility preservation or reconsidering family‐building after new life events such as entering a relationship or experiencing remission [39, 40, 46]. As one Chinese participant recounted:

“After three years, I am now planning to get married. My feelings are different now, so I have started thinking about having children again.” [40]

Yet for those who did pursue pregnancy or fertility treatments, the path was rarely straightforward. Emotional responses were often intense and contradictory—deep joy and amazement alongside anxiety and a sense of being consumed by the process [16, 33, 43, 46, 47, 55]. For some, pregnancy loss brought profound grief, while others reflected on how fertility treatment reactivated feelings from their cancer experience. As one woman reflected:

“Going through infertility treatments is extremely difficult for a woman who has been through breast cancer…it brings back a lot of feelings that I haven't felt in a couple years.” [46]

It was notable that some participants described redefining what fertility meant to them in ways that departed from the sociocultural narratives discussed above [34, 37, 41, 44, 45]. Rather than viewing biological reproduction as essential to a meaningful life, they emphasised alternative sources of identity, such as work, faith, caregiving roles, and personal autonomy [34, 41, 44, 45]. As one participant described, referring to her pursuit of a nursing career:

“That's another way that I can kind of get that ‘caretaking’ feeling.” [34]

For some, the cancer experience became a turning point that prompted reflection on societal expectations and a reconfiguration of future life paths [37, 41, 44]. As one woman said:

“I'm grateful this happened to me at an early age, I've had the opportunity to take stock and take it as a good opportunity to not take life for granted and actually go for the things that you want to go for.” [41]

Others who had been undecided about family‐building prior to cancer described how the experience, paradoxically, helped clarify their priorities [37, 39, 43]. As one survivor put it:

“I had already reached a point where I was basically okay with not having children. The cancer diagnosis just sealed the deal.” [39]

For some, the absence of distress reflected longstanding views about parenthood that predated their cancer diagnosis, rather than the suppression of unresolved concerns [37, 39]. Over time, some participants described a shift from an initial focus on childbearing toward a broader and more fluid understanding of womanhood, as one woman reflected:

“Being a woman is not just having children.” [37]

While this process of redefining reproductive selfhood was present across all studies, its scope differed across cultural contexts. In studies conducted in the UK, US, Canada, Australia, and Mexico, participants' reflections primarily centred on the relationship between motherhood and femininity. In Chinese‐context studies, participants addressed a broader range of topics, including the meaning of marriage, partnership, and the distribution of family responsibilities [33, 44].

#### 3.4.3 Lived Experience Informs the Reimagining of Macro‐Level Care

Participants' accounts in this theme illustrate the bidirectional quality of the macro‐micro interplay: drawing on their experiences of clinical interactions, relational dynamics and personal reflection, participants articulated how macro‐level structures had shaped their reproductive identity negotiation and identified where those structures fell short. Although some participants recalled positive clinical interactions [17, 32, 53], these were described as exceptions rather than the norm. What distinguished these positive encounters was a sense of being listened to and having their fertility concerns treated as legitimate. A consistent message across studies was the desire to be treated as a whole person rather than solely as a cancer patient [32, 35, 36, 38, 52, 53, 54, 59]. As one survivor reflected:

“Treat me as an intelligent woman…it's my personal choice in taking risk, or not taking risk.” [35]

Participants emphasised that health professionals should provide comprehensive information about fertility options without reproducing assumptions based on age, relationship status, or disease progression. They also stressed that such conversations should be clinician‐initiated and ongoing rather than confined to a single consultation [32, 33, 35, 36, 38, 42, 48, 52, 53, 54, 59].

Beyond informational support, participants identified a need for emotional and psychological care extending well beyond the treatment phase, as fertility‐related distress often emerged months or years after diagnosis [16, 32, 41, 49, 53]. Participants called for structured peer support, formalised referral pathways to fertility specialists and counsellors, and the integration of these into standard survivorship care [32, 40, 47, 53, 55, 58, 59]. Notably, some participants observed that the macro‐level discourses shaping reproductive expectations were themselves beginning to shift [42, 43]. One participant in South Korea noted:

“These days, even women in their late twenties or thirties who are not ill voluntarily opt for egg freezing, thinking about having children later. Hearing such stories can be somewhat comforting.” [42]

Taken together, these accounts show that participants' lived experience of structural constraints did not merely produce distress but generated critical insight into how care systems could better support RI across survivorship.

## 4 Discussion and Conclusion

### 4.1 Discussion

This systematic review, guided by Athan's [24] concept of RI, examines how women experienced post‐cancer fertility not as a fixed biological outcome but as a dynamic, ongoing process of identity negotiation shaped by the interplay between macro‐level discourses and structures and micro‐level relational and internal processes.

The macro‐level analysis highlighted the lasting impact of narratives and discourses of biomedical survival, enacted through clinical and informational practices across health and sociocultural systems. Rather than questioning whether survival should take priority, the findings draw attention to how the surrounding discourses and structural conditions produced a clinical environment in which survivors had few opportunities to consider survival and reproduction as anything other than competing priorities. This echoes Zhang et al. [23], who noted the influence of clinicians' attitudes on fertility decision‐making. Our analysis extends this by showing how survivors internalised this framing, coming to perceive their own reproductive concerns as illegitimate or secondary. It is also of note that the absence of verbalised concerns in a clinical interaction cannot be taken as evidence that RI is not being negotiated: many survivors who did not raise fertility concerns with their clinicians had not resolved these concerns but rather had suppressed them. The macro‐level analysis also drew out structural barriers, including financial costs, fragmented referral pathways, and restrictive policies that constrained survivors' reproductive options, particularly for those who were unmarried or financially disadvantaged [42, 43]. Reproductive justice scholarship has argued that reproductive rights include the right to have children and to parent with adequate institutional support [63]. Given the involuntary nature of cancer‐related fertility disruption, these findings highlight the need to review how policy frameworks in different national contexts account for the reproductive circumstances of cancer survivors, and whether context‐specific responses warrant further development.

These macro‐level discourses were not passively received but actively reproduced within intimate relationships and participants' internal processes at the micro level. The scope of this relational reproduction differed across cultural contexts: studies conducted in Australia, Canada, Mexico, the UK and the US primarily captured pressure within intimate partnerships [17, 37, 38, 39, 41, 43], whereas data from China and Japan pointed to a broader, collective dimension in which fertility decisions were negotiated within extended family structures. At the same time, the analysis revealed survivors' agency in (re)working their reproductive selfhood. While existing reviews have emphasised distress and passive coping [10, 21, 23], our findings indicate that some participants actively reinterpreted and at times resisted the sociocultural frameworks within which their reproductive identities had been formed. Scholarship on gender and reproduction also has shown that women facing reproductive disruption may exercise agency by strategically repositioning themselves within or against dominant narratives of motherhood and femininity [64, 65]. The RI framework is distinctive in its capacity to hold both dimensions, recognising the structural constraints that limit reproductive options alongside the developmental agency through which survivors negotiate their reproductive selfhood.

Our analysis showed that participants redefined fertility in symbolic, social, and relational terms that extended well beyond the question of whether to bear children. This aligns with Athan's [24] argument that RI cannot be reduced to the pursuit of biological parenthood: an individual who does not have children may nonetheless relate to themselves in parental terms. These findings suggest what is currently recognised as a reproductive concern in survivorship care may need to become more expansive and responsive to the diverse and evolving ways in which survivors relate to their reproductive selves. More broadly, the post‐cancer fertility experiences documented in this review may be understood as one manifestation within the wider spectrum of RI, contributing lived empirical detail to a concept that Athan [24] proposed as a preliminary theoretical formulation.

## 5 Limitations

This review has several limitations. The inclusion of studies published between 2010 and 2026 introduced some historical heterogeneity, though the consistency of core concerns across this period suggests relative stability in the macro‐level conditions shaping survivors' reproductive experiences. The restriction to English‐ and Chinese‐language publications may have excluded relevant research in other languages. The included studies covered a wide range of cancer diagnoses, treatment modalities, and survivorship stages, and the review did not examine whether these produced distinct patterns of RI negotiation. Although overlapping samples from two shared research projects were accounted for in participant counts, including four articles from these projects may have influenced the weight assigned to certain themes, and some studies that included partners or healthcare providers alongside female survivors may have introduced contextual variation despite extracting only female survivor data. The age criterion of 18–50 may also have excluded relevant experiences beyond this range. While all studies met moderate‐to‐high quality thresholds on the CASP appraisal, limited reporting of reflexivity across most included studies means that original researchers' positionality in relation to participants' fertility experiences is largely unknown. Finally, differences identified across geographical locations were not explored in detail, and the contextual patterns observed in the East Asian studies were drawn from a limited number of locations and should be treated as preliminary. Future research could usefully examine how patterns of RI negotiation vary across different sociocultural and institutional contexts.

## 6 Conclusion

This review, guided by Athan's [24] concept of reproductive identity, demonstrates that post‐cancer fertility is not a fixed medical status but a dynamic, ongoing process of identity negotiation shaped by the interplay between macro‐level discourses and structures and micro‐level relational and internal processes. By positioning post‐cancer fertility within the broader spectrum of reproductive identity, this review offers an integrative, developmental lens on it and highlights the practical value of embedding reproductive identity literacy into survivorship care.

## 7 Practical Implications

The findings carry several implications for the design of survivorship care services. First, care service providers should recognise that post‐cancer fertility involves ongoing developmental identity negotiation and should proactively provide structured touchpoints for fertility‐related conversations across the survivorship trajectory. In doing so, it is important to recognise the potential of RI literacy to be used as a framework for initiating conversations about fertility without assumptions based on age, relationship status, or existing children. Second, the structural barriers documented in this review underscore the importance of establishing formalised referral pathways that integrate oncology with reproductive endocrinology, fertility counselling, and psychosocial support within a coordinated care model, alongside further policy discussion about equitable access to fertility preservation for cancer survivors. Third, consistent with Athan's [24] framing of RI as existing on a non‐binary spectrum, survivorship care planners are encouraged to create space for diverse reproductive trajectories that may or may not include biological parenthood.

## Author Contributions

**Yijun Wang:** conceptualisation, data curation, methodology, formal analysis, writing – original draft, visualisation. **Margo Louise Turnbull:** conceptualisation, supervision, methodology, formal analysis, writing – original draft, writing – review and editing. **Jeffry Oktavianus:** formal analysis, writing – review and editing. **Xiaoyan I. Wu:** methodology, formal analysis, writing – review and editing.

## Ethics Statement

This is a systematic review. Ethics approved is not applicable.

## Conflicts of Interest

The authors declare no conflicts of interest.

## Supporting information


Supporting File 1

Supporting File 2

Supporting File 3

Supporting File 4

## Data Availability

Data sharing not applicable to this article as no datasets were generated or analysed during the current study.
