# Supplementary material for: Negotiating Reproductive Identity After Cancer: A Qualitative Systematic Review of Female Survivors' Post‐Treatment Fertility Experiences
Source: Health Expect. 2026 Oct 21;29(5):e70811. doi: 10.1111/hex.70811 (PMC13686586; doi:10.1111/hex.70811)
Supplement: Supplementary file 1 [file HEX-29-e70811-s001.docx]

**CASP SCORES**

*A score of 1 indicated that the item had been thoroughly discussed in the manuscript; a score of 0.5 meant it was unclear or there was limited discussion of the item; and a score of 0 indicated that the item was not discussed or was inadequate to the research design. Overall scores >9 were deemed high quality; scores between 7 and 9 were deemed moderate quality; and scores <7 were low quality.*

| **Article** | **Lloyd et al (2014)** | **Dryden et al (2014)** | **Ruddy et al (2015)** | **Hubbeling et al (2018)** | **Benedict et al (2020)** | **Benedict et al (2021)** | **Sobota & Ozakinci (2021)** | **Vanstone et al (2021)** | **Sira et al (2024)** | **Rowlands et al (n.d.)** | **Gorman et al (2011)** | **Lee et al (2011)** | **Gorman et al (2012)** | **Kirkman et al (2013)** | **Corney & Swinglehurst (2014)** |
| --- | --- | --- | --- | --- | --- | --- | --- | --- | --- | --- | --- | --- | --- | --- | --- |
| *Q1 Was there a clear statement of the aims of the research?* | 1 | 1 | 1 | 1 | 1 | 1 | 1 | 1 | 1 | 1 | 1 | 1 | 1 | 1 | 1 |
| *Q2 Is a qualitative methodology appropriate?* | 1 | 1 | 1 | 1 | 1 | 1 | 1 | 1 | 1 | 1 | 1 | 1 | 1 | 1 | 1 |
| *Q3 Was the research design appropriate to address the aims of the research?* | 1 | 1 | 1 | 1 | 1 | 1 | 1 | 1 | 1 | 1 | 1 | 1 | 1 | 1 | 1 |
| *Q4 Was the recruitment strategy appropriate to the aims of the research?* | 1 | 1 | 1 | 1 | 1 | 1 | 1 | 1 | 1 | 1 | 1 | 1 | 1 | 1 | 1 |
| *Q5 Was the data collected in a way that addressed the research issue?* | 1 | 1 | 1 | 1 | 1 | 1 | 1 | 1 | 1 | 1 | 1 | 1 | 1 | 1 | 1 |
| *Q6 Has the relationship between researcher and participants been adequately considered?* | 0.5 | 0.5 | 0.5 | 1 | 0.5 | 0.5 | 1 | 0.5 | 0.5 | 1 | 0.5 | 0.5 | 0.5 | 0.5 | 0.5 |
| *Q7 Have ethical issues been taken into consideration?* | 0.5 | 1 | 1 | 1 | 1 | 1 | 1 | 1 | 1 | 1 | 1 | 0.5 | 0.5 | 1 | 1 |
| *Q8 Was the data analysis sufficiently rigorous?* | 1 | 1 | 1 | 1 | 1 | 1 | 1 | 1 | 1 | 1 | 1 | 1 | 1 | 1 | 1 |
| *Q9 Is there a clear statement of findings?* | 1 | 1 | 1 | 1 | 1 | 1 | 1 | 1 | 1 | 1 | 1 | 1 | 1 | 1 | 1 |
| *Q10 How valuable is the research?* | 1 | 1 | 1 | 1 | 1 | 1 | 1 | 1 | 1 | 1 | 1 | 1 | 1 | 1 | 1 |
| **Score** | **9** | **9.5** | **9.5** | **10** | **9.5** | **9.5** | **10** | **9.5** | **9.5** | **10** | **9.5** | **9** | **9** | **9.5** | **9.5** |

| **Article** | **Corney et al (2014)** | **Komatsu et al (2014)** | **Standen et al (2020)** | **Gorman et al (2022)** | **ZhouJie et al (2022)** | **Qiu Jiajia et al (2024)** | **Li Xia & Qiang Wanmin (2016)** | **TANG Lin et al (2020)** | **AN Pei et al (2022)** | **Wang Xiaoyuan et al (2021)** | **Yao Hong et al (2024)** | **Singh et al (2026)** | **Han et al (2025)** | **Banser et al (2025)** | **Xu et al (2024)** |
| --- | --- | --- | --- | --- | --- | --- | --- | --- | --- | --- | --- | --- | --- | --- | --- |
| *Q1 Was there a clear statement of the aims of the research?* | 1 | 1 | 1 | 1 | 1 | 1 | 1 | 1 | 1 | 1 | 1 | 1 | 1 | 1 | 1 |
| *Q2 Is a qualitative methodology appropriate?* | 1 | 1 | 1 | 1 | 1 | 1 | 1 | 1 | 1 | 1 | 1 | 1 | 1 | 1 | 1 |
| *Q3 Was the research design appropriate to address the aims of the research?* | 1 | 1 | 1 | 1 | 1 | 1 | 1 | 1 | 1 | 1 | 1 | 1 | 1 | 1 | 1 |
| *Q4 Was the recruitment strategy appropriate to the aims of the research?* | 1 | 1 | 1 | 1 | 1 | 1 | 1 | 1 | 1 | 1 | 1 | 1 | 1 | 1 | 1 |
| *Q5 Was the data collected in a way that addressed the research issue?* | 1 | 1 | 1 | 1 | 1 | 1 | 1 | 1 | 1 | 1 | 1 | 1 | 1 | 1 | 1 |
| *Q6 Has the relationship between researcher and participants been adequately considered?* | 0.5 | 0.5 | 0.5 | 0.5 | 0.5 | 0.5 | 0.5 | 0.5 | 0.5 | 0.5 | 0.5 | 0.5 | 0.5 | 1 | 1 |
| *Q7 Have ethical issues been taken into consideration?* | 1 | 1 | 1 | 1 | 0.5 | 1 | 1 | 1 | 0.5 | 0.5 | 0.5 | 1 | 1 | 1 | 1 |
| *Q8 Was the data analysis sufficiently rigorous?* | 1 | 1 | 1 | 1 | 1 | 1 | 1 | 1 | 1 | 1 | 1 | 1 | 1 | 1 | 1 |
| *Q9 Is there a clear statement of findings?* | 1 | 1 | 1 | 1 | 1 | 1 | 1 | 1 | 1 | 1 | 1 | 1 | 1 | 1 | 1 |
| *Q10 How valuable is the research?* | 1 | 1 | 1 | 1 | 1 | 1 | 1 | 1 | 1 | 1 | 1 | 1 | 1 | 1 | 1 |
| **Score** | **9.5** | **9.5** | **9.5** | **9.5** | **9** | **9.5** | **9.5** | **9.5** | **9** | **9** | **9** | **9.5** | **9.5** | **10** | **10** |

**SUMMARY OF CASP SCORES**

**Number of Answers Across All Included Studies**

|  | **CASP question** | **Yes** | **Unclear** | **No** |
| --- | --- | --- | --- | --- |
|  | ***RIGOUR—Are the results valid?*** | | | |
| Q1 | Was there a clear statement of the aims of the research? | 30 | 0 | 0 |
| Q2 | Is a qualitative methodology appropriate? | 30 | 0 | 0 |
| Q3 | Was the research design appropriate to address the aims of the research? | 30 | 0 | 0 |
| Q4 | Was the recruitment strategy appropriate to the aims of the research? | 30 | 0 | 0 |
| Q5 | Was the data collected in a way that addressed the research issue? | 30 | 0 | 0 |
| Q6 | Has the relationship between researcher and participants been adequately considered? | 5 | 25 | 0 |
|  | ***CREDIBILITY—What are the results?*** | | | |
| Q7 | Have ethical issues been taken into consideration? | 23 | 7 | 0 |
| Q8 | Was the data analysis sufficiently rigorous? | 30 | 0 | 0 |
| Q9 | Is there a clear statement of findings? | 30 | 0 | 0 |
|  | ***RELEVANCE—Will the results help locally?*** | | | |
| Q10 | How valuable is the research? | 30 | 0 | 0 |
