# Supplementary material for: Negotiating Reproductive Identity After Cancer: A Qualitative Systematic Review of Female Survivors' Post‐Treatment Fertility Experiences
Source: Health Expect. 2026 Oct 21;29(5):e70811. doi: 10.1111/hex.70811 (PMC13686586; doi:10.1111/hex.70811)
Supplement: Supplementary file 2 [file HEX-29-e70811-s002.docx]

## Coding Guideline

**Systematic Review Title:** *Negotiating Reproductive Identity After Cancer: A Qualitative Systematic Review of Female Survivors’ Post-Treatment Fertility Experiences*

**Research Question: (a) How are macro-level narratives reproduced in female cancer survivors’ experiences of post-treatment fertility? (b) What micro-level relational and personal processes are evident in experiences of reproductive identity shaping?**

## Coding in NVivo

Note: You’re using inductive coding only for the **findings section** of the articles.

In this step, you will code the data directly under six pre-defined categories, developed based on pilot open coding conducted by us. **You do not need to create new categories.** If a segment of text does not clearly fit into one of the six categories, please place it under a temporary **“Other”** node for later discussion. If a passage fits more than one, double code it.

Focus on coding content that reflects:

•	Participant reflections and lived experiences

•	Interpretations and meaning making related to fertility

•	Social, cultural, emotional, and relational aspects of survivorship

**6 Main Coding Categories**:

- **Fertility & Medical Interactions**

Interactions with healthcare professionals (e.g., oncologists, fertility specialists, nurses) related to fertility during and after cancer treatment. It includes both the content and quality of medical communication, as well as survivors’ expectations and unmet needs in these interactions.

- **Fertility & Emotions**

Emotional and psychological responses related to fertility experiences: Grief, fear, anxiety, hope, guilt, or emotional relief.

- **Fertility & Identity of Self**

How fertility concerns impact self-concept, womanhood, motherhood, and life trajectory: Biographical disruption, identity reconstruction, self-worth

- **Fertility & Relationships (Partner, Family, Peers)**

How fertility issues affect romantic, familial, and social relationships: Partner dynamics, dating concerns, family expectations.

- **Fertility & Values, Beliefs**

Survivors’ attitudes, values, and beliefs regarding fertility and reproduction after cancer: Reproductive decision-making (e.g., pursuing or foregoing pregnancy); Personal or cultural values around parenthood; Moral, spiritual, or existential beliefs affecting fertility choices.

- **Event of pregnancy**

Narratives related to pregnancy after cancer: Attempts, successes and failures.

## Important Reminders

•	If you identify data that does not clearly fit the six main categories, please create a temporary category called “Other” and flag it for group discussion.

•	If a passage fits more than one, double code it.

•	Use NVivo memos to document: Unclear passages; Emerging patterns; Reflections on fit between codes and data

•	We will review all “Other” items and proposed refinements in the next synthesis meeting.

•	Stay grounded in the participant voice and the RQ.

•	If you encounter challenges interpreting Chinese-language content or applying English category definitions, please reach out so we can adapt together.
