# Supplementary material for: Negotiating Reproductive Identity After Cancer: A Qualitative Systematic Review of Female Survivors' Post‐Treatment Fertility Experiences
Source: Health Expect. 2026 Oct 21;29(5):e70811. doi: 10.1111/hex.70811 (PMC13686586; doi:10.1111/hex.70811)
Supplement: Supplementary file 3 [file HEX-29-e70811-s004.docx]

| **Search Criteria** | **Terminology** |
| --- | --- |
| **Cancer** | cancer OR neoplasm* |
| **Fertility issues** | fertilit* OR reproductiv* |
| **Female** | female* OR wom?n |
| **Psychological and social factors** | psycholog* OR "mental health" OR anxiety OR "well-being" OR distress OR coping OR social OR "family" OR peer* |
| **Survivorship period** | survivorship OR "post-treatment" OR "long-term survivorship" OR "life after cancer" OR "cancer surviv* |

Appendix A English & Chinese Search terms

| **Search Criteria** | **Terminology** |
| --- | --- |
| 癌症 | 癌症 OR 瘤 OR 恶性肿瘤 |
| 生育  女性 | 生育忧虑 OR 生育抑郁 OR 生育忧郁 OR 生育焦虑 OR 生育担忧  女性 |
